# Prevalence and prognostic value of FBXO11 expression in patients with clear cell renal cell carcinoma

**DOI:** 10.1186/s12885-019-5736-8

**Published:** 2019-06-03

**Authors:** Bo Fan, Wei Wang, Xianping Zhang, Min Sun, Xiaogang Wang, Zhiqi Chen, Wankai Liu, Qun Wang, Na Yu, Xiancheng Li

**Affiliations:** 1 Department of Urology, Second Affiliated Hospital of Dalian Medical University, Dalian, 116011 Liaoning Province China; 2 Department of General Surgery, Taihe Hospital of Hubei University of Medicine, Shiyan, 442000 Hubei Province China

**Keywords:** FBXO11, Renal cell carcinoma, Prognosis, Nomogram

## Abstract

**Background:**

FBXO11, a member of the F-box protein family, regulates the cell-cycle by promoting the degradation of Bcl-6 and p53. This protein has been implicated in the progression of several cancers, including renal cell carcinoma (RCC). The aim of this study was to determine the prognostic role of FBXO11 in the clinical outcome of RCC patients.

**Methods:**

FBXO11 mRNA expression was analysed in normal and RCC tissue microarrays of the Oncomine database. In addition, the in situ expression levels of stromal FBXO11 protein were assessed in primary RCC tissues from 227 patients (training and validation cohorts) using immunohistochemistry (IHC). Kaplan Meier and Cox regression analyses were used to determine the association between FBXO11 expression and cliniopathological factors. A nomogram was established using the significant prognostic factors to predict overall survival (OS) of RCC patients after one, three and 5 years.

**Results:**

In the Oncomine database, FBXO11 mRNA levels were lower in normal tissues than in cancer tissues, including clear cell renal cell carcinoma (ccRCC), papillary renal cell carcinoma (pRCC), hereditary ccRCC, non-hereditary ccRCC, VHL mutant ccRCC and VHL wild-type ccRCC. In addition, FBXO11 expression was also significantly higher in metastatic kidney cancer than in primary cancer. Immunohistochemical analysis reported that 57.3% (86 of 150) of the training cohort and 57.1% (44 of 77) of the validation cohort were scored as having high FBXO11 staining density. FBXO11 expression was significantly associated with Fuhrman grade (*p* = 0.003), UISS score (*p* = 0.021) and age (*p* = 0.048) in the training cohort. Furthermore, Kaplan-Meier survival analysis showed that higher FBXO11 levels, T stage, UISS scores and SSIGN score were associated with poor OS in ccRCC patients. Multivariate Cox analysis demonstrated that higher FBXO11 levels and higher UISS score were independent prognostic indicators for OS. Nomogram, calibration plots, AUC values and the C-index showed that the predictive accuracy of conventional prognostic models, including UISS score and SSIGN score, was improved when FBXO11 expression was added.

**Conclusions:**

FBXO11 expression was closely related to RCC malignancy and poor prognosis, indicating its potential as a prognostic marker as well as a therapeutic target for RCC.

## Background

Renal cell carcinoma (RCC) is the third most common cancer of the urinary system and accounts for approximately 2–3% of all cancers [1, 2]. An estimated 65,340 new cases of kidney cancer and renal pelvic cancer, and 14,970 deaths relating to these diseases were projected for 2018 in the United States alone [3]. Despite advanced diagnostic methods, one-third of clear cell RCC (ccRCC) patients are diagnosed when the cancer has already metastasized, and indicates poor prognosis. Although RCC-related mortality has decreased due to the advent of minimally invasive surgery, targeted therapy, etc., it is still a major health concern in Asia [4, 5]. The prognostic factors for RCC [6–9] include anatomical characteristics (tumour size, renal capsular invasion, venous invasion, etc.), histological features (tumour grade, RCC subtype, sarcomatoid features, etc.) and clinicopathological features (performance status, local symptoms, neutrophil/lymphocyte ratio, etc.). However, the predictive accuracy of the current prognostic systems associated with these markers needs to be improved. [8–10]. Therefore, the molecular mechanisms underlying the metastasis and progression of kidney cancer need to be elucidated in order to improve its prognosis.

The FBXO11 protein consists of ~ 843 amino acids with a predicted molecular weight of 94 kDa, and the encoding gene is located on chromosome 2p21. As a member of the SKP1-CUL1-F-box ubiquitin ligase complex (wherein the FBOX family members act as substrate adaptors), FBXO11 targets proteins for ubiquitination and proteosomal degradation [11, 12] and is therefore relevant in several diseases. For example, the FBXO11 gene is downregulated in vitiligo patients, and may be related to the loss of the pigment-producing melanocytes [12, 13]. Furthermore, FBXO11 also acts as a nedd8 ligase for the tumour suppressor gene *TP53* by mediating the NEDDylation of p53 protein [13, 14], and transcriptionally inactivating *TP53*. FBXO11 also promotes the ubiquitin-driven degradation of Snail, a transcription factor mediating the epithelial-mesenchymal transition (EMT) during cancer progression and metastasis [15]. Specifically, in breast cancer, FBXO11 inhibits metastatic progression by targeting the Snail protein for degradation and blocking Snail-induced EMT [12]. However, any potential clinical significance of FBOX11 in ccRCC is currently unknown, and no studies so far have investigated the correlation between FBXO11 expression and the clinico-pathological features and prognostic outcomes of ccRCC patients.

In the present study, we first examined FBXO11 expression levels in normal and malignant renal tissues using datasets available through ONCOMINE, a web-based cancer microarray database. In addition, we also analysed the in situ expression of FBXO11 in 227 ccRCC tissues (training and validation cohorts) and 40 normal kidney tissues. The association between FBXO11 expression levels and prognostic clinical outcomes was analysed using Cox regression models. Finally, a nomogram model was established using the significant prognostic factors to predict overall survival (OS).

## Methods

### Analysis of Oncomine data

The expression pattern of FBXO11 in kidney cancer tissues was determined using the Oncomine database (https://www.oncomine.org). Briefly, the database was searched for the FBXO11 gene and the results were filtered by selecting kidney cancer vs. normal analysis. Box charts were used to display the data, and *P*-values for each group were calculated using Student’s t-test. The specifics of normalization and statistical calculations are listed in the database program.

### Patients and samples

All patients (including those in the training and validation cohorts) who were pathologically diagnosed with ccRCC at the Second Affiliated Hospital of Dalian Medical University were included in the study. Tumour tissues were collected after the first surgery, which included radical nephrectomy or nephron-sparing surgery. The clinical and pathological baseline data, including age, gender, TMN stage, tumour grade, maximum diameter of tumour, lymph metastasis, sarcomatosis and tumour necrosis were recorded. TMN staging and tumour grade was determined according to the 2004 WHO/ISUP classification. Forty normal kidney tissues were procured from surgically treated patients with renal pelvic carcinoma or ureteral urothelial carcinoma. This study was approved by the Ethics Committee of Second Affiliated Hospital of Dalian Medical University.

### Immunohistochemistry (IHC)

Tissue sections were stained by standard IHC protocols as previously described [16]. Briefly, after overnight deparaffinization in dimethylbenzene and rehydration in an alcohol gradient, the sections were heated in antigen retrieval buffer at 120 °C for 5 min. After endogenous peroxidase was quenched with 3% hydrogen peroxide for 30 min, the sections were incubated at 4 °C for 24 h with primary antibodies against FBXO11 (ab110965, Abcam). The samples were then incubated with biotinylated secondary antibodies and horseradish peroxidase labelled avidin, and the colour was developed using DAB (ZSGBBIO, Beijing, China). The stromal FBXO11 expression level was analysed by two investigators who were blinded to the clinical and prognostic data. The total staining intensity was scored as: 0 (no staining), 1 (weak staining), 2 (moderate staining) and 3 (strong staining), and the percentage of positive cells was scored as: 0 (0%), 1 (1 to 50%), 2 (51 to 80%), and 3 (> 80%). The overall FBXO11 expression was calculated by multiplying the score of the percentage of positive cells with the intensity score: 0 to 3 was defined as low expression, and > 3 was defined high expression.

### Statistical analysis

Statistical analysis was performed with SPSS 13.0 (SPSS Inc., Chicago, USA). The baseline data were compared between groups using the χ^2^ or Fisher’s exact test. Correlations between clinicopathological variables and FBXO11 expression were assessed by Spearman’s rank correlation test. Overall survival (OS) was defined from the onset of treatment to last follow-up (censored) or death (event), estimated by the Kaplan Meier method, and compared by the log-rank test. A multivariable Cox regression model was used to estimate the independent statistical significance of the different variables. A nomogram and calibration plot for OS based on multivariable analysis were constructed with R software (R Foundation for Statistical Computing, Vienna, Austria). The accuracy of clinical outcomes and the discriminatory ability of prognostic models were evaluated by the concordance index (C-index) and Akaike information criterion (AIC) value respectively. A *P*-value< 0.05 was considered statistically significant.

## Results

### Higher FBXO11 mRNA levels were observed in kidney cancer tissues

**Fig. 1:**
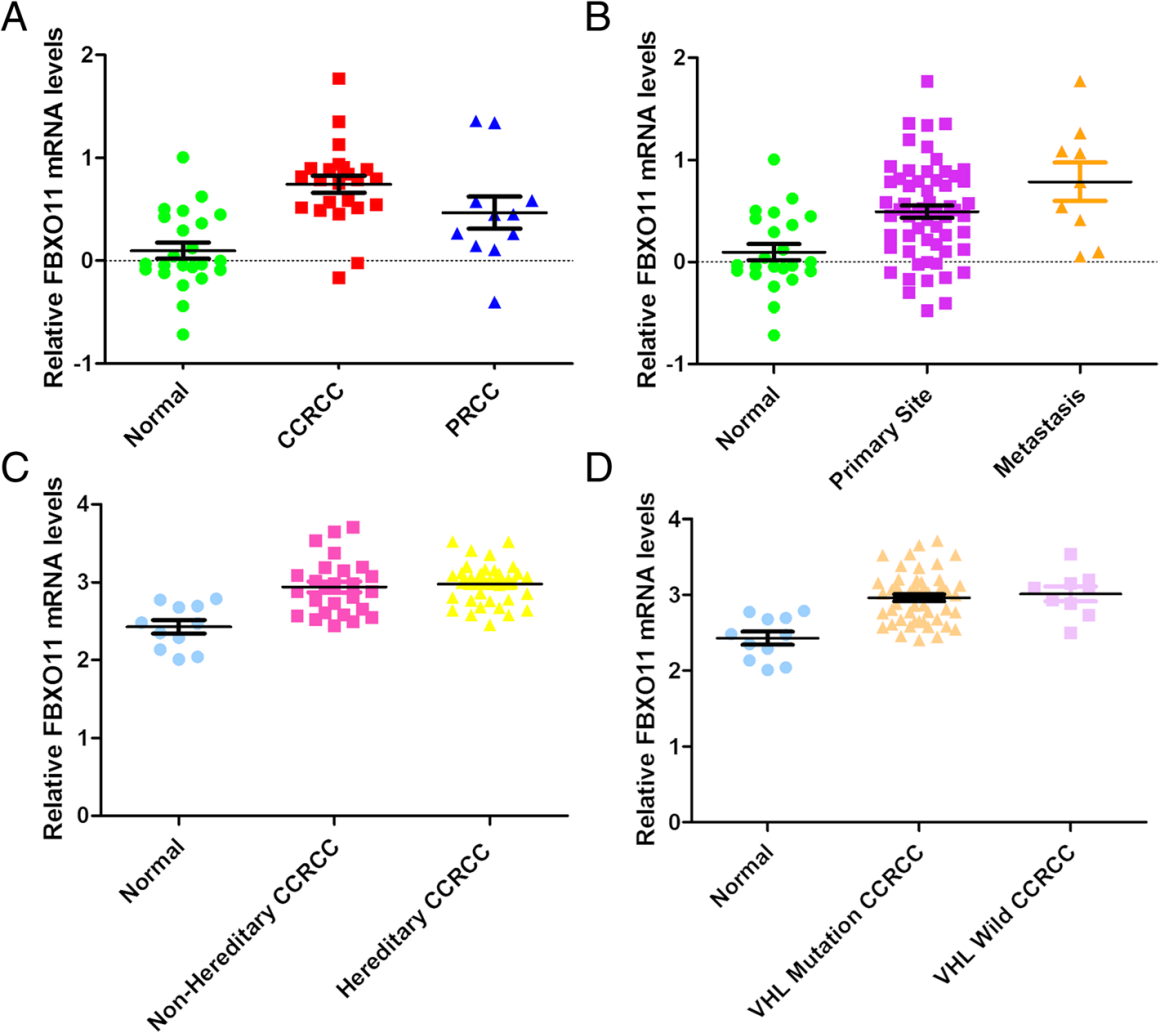
FBXO11 expression in ONCOMINE renal tissue datasets. **a** FBXO11 expression stratified by tissue type (cancer vs. normal) in the Jones Renal microarray dataset. **b** FBXO11 expression stratified by metastasis status (metastasis vs. primary) in the Jones Renal microarray dataset. **c** FBXO11 expression stratified by hereditary characteristics (hereditary vs. non- hereditary) in the Beroukhim Renal microarray datasets. **d** FBXO11 expression stratified by VHL mutation status (mutant vs. wild-type) in the Beroukhim Renal microarray datasets

We analysed the FBXO11 levels in the Jones Renal microarray dataset from the Oncomine (http://www.oncomine.org/) dataset [17]. FBXO11 expression was significantly lower in normal tissues than in cancer tissues (23 ccRCC and 11 pRCC samples) (*P* < 0.001, *F* = 14.03, Fig. 1a). In addition, FBXO11 expression was significantly higher in the metastatic tissues than in the primary cancer tissues (*P* = 0.002, *F* = 9.474, Fig. 1b). The data of the metastatic samples are also derived from the Jones Renal microarray dataset from the Oncomine (http://www.oncomine.org/) dataset. In the study of the Jones data, metastatic tumours included distant and nodal metastatic ccRCC. The Beroukhim Renal microarray datasets [18] showed that FBXO11 expression in normal tissue was notably lower than that in both non-hereditary and hereditary ccRCC (*P* < 0.001, *F* = 13.61, Fig. 1c), as well as than that in VHL mutant and wild-type ccRCC (*P* < 0.001, *F* = 13.30, Fig. 1d).

### Association of FBXO11 expression with clinico-pathological factors

**Fig. 2:**
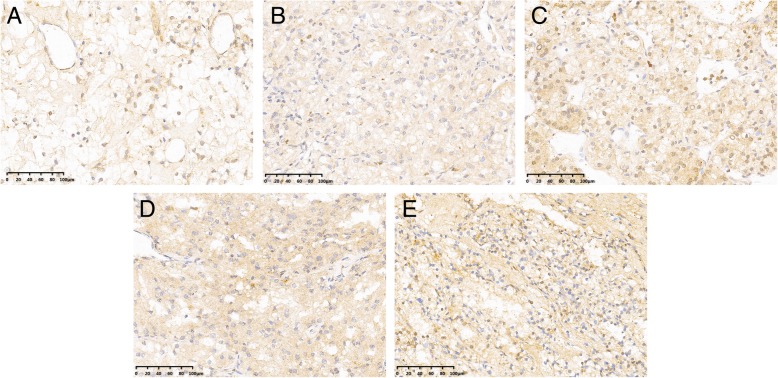
Representative immunohistochemical staining of FBXO11 expression in RCC specimens at 200 magnification**. a** No expression of FBXO11 in normal kidney tissue (0% staining) as a contrast. **b** Low expression of FBXO11 in RCC tissue with Fuhrman 1 (15% staining). **c** High expression of FBXO11 in RCC tissue with Fuhrman 2 (50% staining). **d** High expression of FBXO11 in RCC tissue with Fuhrman 3 (80% staining). **e** High expression of FBXO11 in RCC tissue with Fuhrman 4 (100% staining). A high expression level of FBXO11 appears as brown yellow or brown particles

To determine the clinical relevance of FBXO11 expression in ccRCC, we evaluated the in situ levels in tumour tissues resected from patients in the two cohorts. (Fig. 2). Based on staining intensity, we divided the 227 ccRCC patients into the low-FBXO11 (*n* = 97) and high-FBXO11 (*n* = 130) groups. Positive FBXO11 expression was observed in 57.3% (130/227) of the ccRCC, tissues but only 17.5% (7/40) of the normal kidney tissues. A total of 57.3% (86 of 150) in the training cohort and 57.1% (44 of 77) in the validation cohort of tumour tissues weas scored as having high FBXO11 staining density. The correlations between FBXO11 expression and major clinico-pathological features, including age, gender, tumour size, Fuhrman grade, T stage, sarcomatoid status, lymph node status, distant metastasis and types of surgery are summarized in Table 1. Moreover, FBXO11 expression was significantly associated with Fuhrman grade (*p* = 0.003), UISS score (*p* = 0.021) and age (*p* = 0.048) in the training cohort was significantly related to UISS score (*p* = 0.012) in the validation cohort.

**Table 1:** Distribution of characteristics of two independent patient cohorts with RCC by FBXO11 expression

Patient characteristics	Training cohort (150 patients)		Total	*P* Value	Validation cohort (77patients)		Total	*P* Value
	Low	High			Low	High		
Gender				0.227				0.644
Male	41	63	104		17	25	42	
Female	23	23	46		16	19	35	
Age, years				**0.048**				0.099
<57	38	37	75		19	17	36	
≥57	26	49	75		14	27	41	
Tumor size				0.738				0.394
≤4 cm	30	35	65		15	14	29	
4 cm–7 cm	24	35	59		13	24	37	
≥7 cm	10	16	26		5	6	11	
Fuhrman grade				**0.003**				0.111
I-II	47	39	86		22	19	41	
III	14	37	51		8	16	24	
IV	3	10	13		3	9	12	
Pathologic stage				0.065				0.291
T1	58	66	124		28	29	57	
T2	5	13	18		2	8	10	
T3	1	7	8		2	5	7	
T4	0	0	0		1	2	3	
Lymph node status				0.764				0.423
No	62	84	146		31	39	70	
N1- Nx	2	5	4		2	5	7	
Distant metastasis				0.131				0.894
Absent	64	83	147		31	41	72	
Present	0	3	3		2	3	5	
Sarcomatoid				0.846				0.894
Absent	59	80	139		31	41	72	
Present	5	6	11		2	3	5	
Type of surgery				0.395				0.836
Radical nephrectomy	42	62	104		21	29	50	
Partial nephrectomy.	22	24	46		12	15	27	
UISS score				**0.021**				**0.012**
1	52	52	104		26	20	46	
2	11	29	40		7	23	30	
3	1	5	6		0	1	1	
SSIGN score				0.205				0.070
0–3	58	70	128		27	26	53	
4–7	6	14	20		3	13	16	
≥8	3	5	8		3	5	8	

Bold values are considered statistically significant (*p* < 0.05)

### High FBXO11 expression corresponded to poor prognosis in ccRCC

**Fig. 3:**
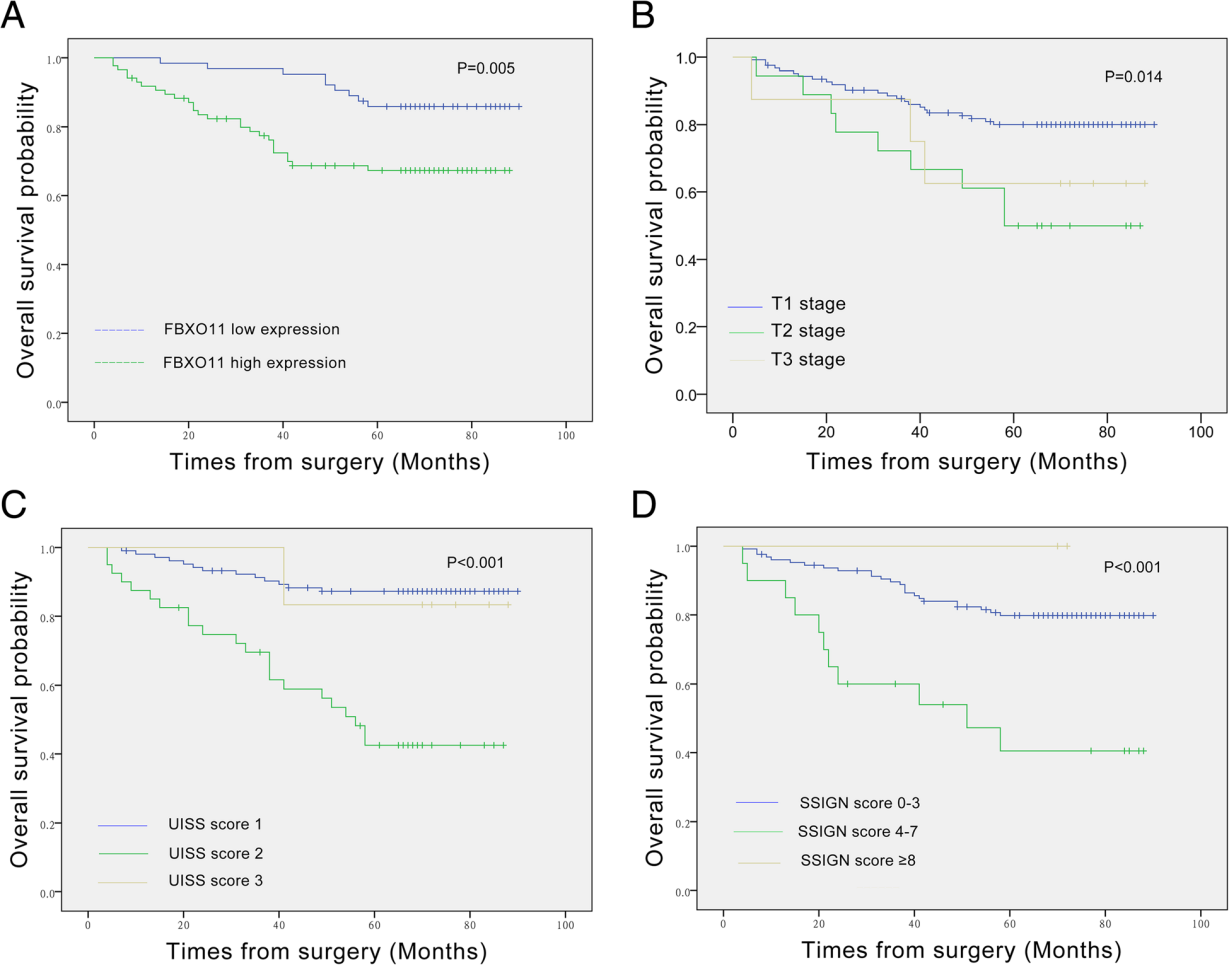
Generated survival curves for training cohort patients generated using the Kaplan-Meier method and log-rank tests. **a** Overall survival curves for low FBXO11 expression (green line) and high FBXO11 expression (blue line), indicating that a high level of FBXO11 protein was significantly associated with worse survival in the IHC cohort. **b** Overall survival curves for T1 stage (blue line), T2 stage (green line, 2), and T3 stage (light yellow line), suggesting that RCC patients with T3 stage disease had worse survival than individuals with T1 stage or T2 stage disease. **c** Overall survival curves for UISS score 1 (blue line), UISS score 2 (green line) and UISS score 3 (light yellow line), illustrating that RCC patients with a UISS score of 1 had better survival than individuals with a UISS score 2 or 3. **d** Overall survival curves for SSIGN score 0–3 (blue line), SSIGN score 4–7 (green line) and SSIGN score ≥ 8 (light yellow line), demonstrating that RCC patients with a lower SSIGN score had better overall survival than individuals with a high SSIGN score

**Fig. 4:**
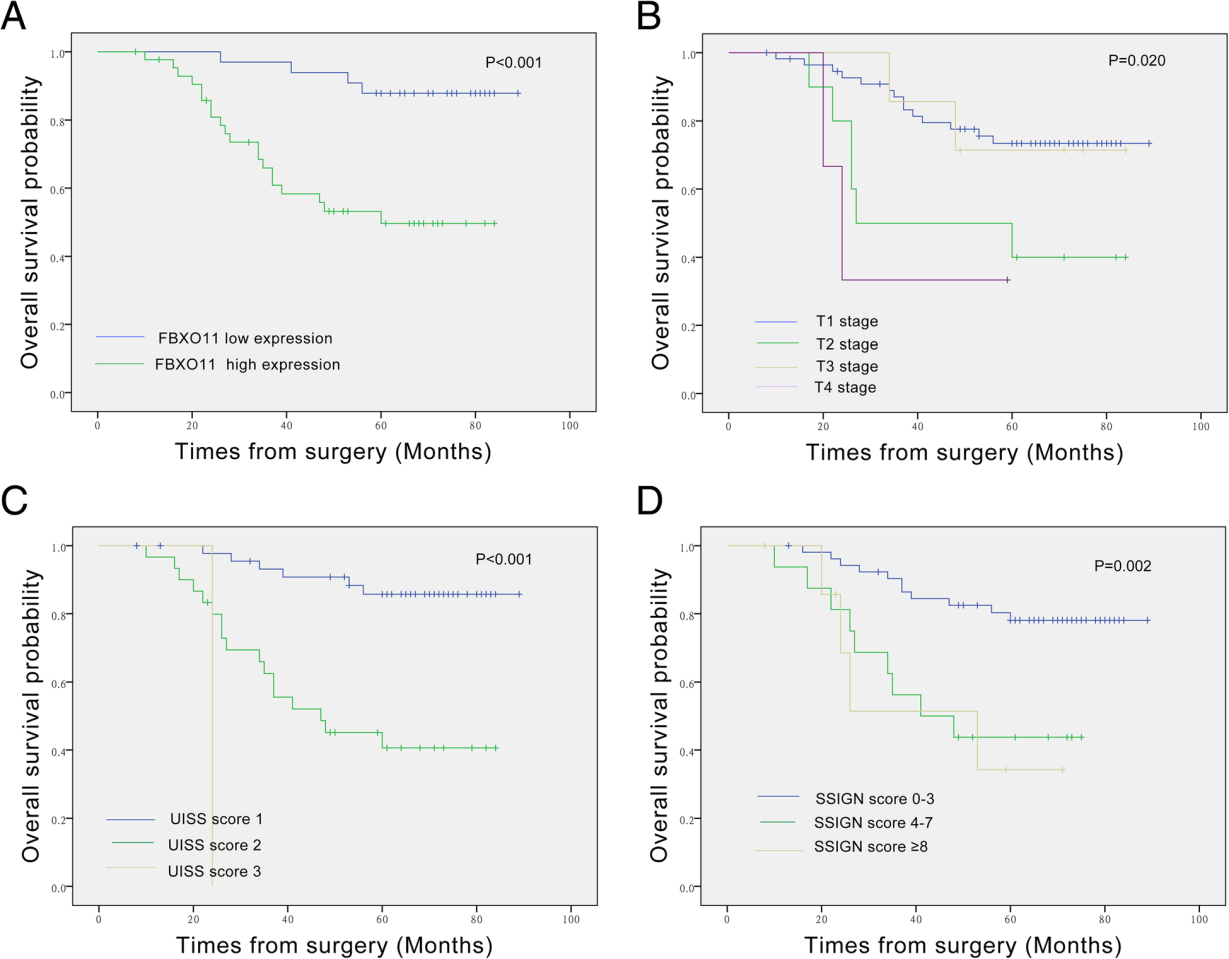
Generated survival curves for validation cohort patients using the Kaplan-Meier method and log-rank tests. **a** Overall survival curves for low FBXO11 expression (green line) and high FBXO11 expression (blue line), indicating high level of FBXO11 protein was significantly associated with worse survival in the IHC cohort. **b** Overall survival curves for T1 stage (blue line), T2 stage (green line, 2), and T3 stage (light yellow line), suggesting that RCC patients with T3 stage had worse survival than individuals with T1 stage or T2 stage. **c** Overall survival curves for UISS score 1 (blue line), UISS score 2 (green line) and UISS score 3 (light yellow line), illustrating that RCC patients with a UISS score of 1 had better survival than individuals with a UISS score of 2 or 3. **d** Overall survival curves for SSIGN score 0–3 (blue line), SSIGN score 4–7 (green line) and SSIGN score ≥ 8 (light yellow line), demonstrating that RCC patients with a lower SSIGN score had better survival than individuals with a high SSIGN score

The Kaplan Meier survival curve showed that higher FBXO11 levels, T stage, UISS score and SSIGN score were associated with poor OS not only in the training cohort, but also in the validation cohort (Fig. 3a-d and Fig. 4a-d). After univariate and multivariate analysis under the Cox proportional hazard model, higher FBXO11 expression levels were found to be independent prognostic indicators in not only the training cohort (HR =2.381; 95% CI = 1.089–5.205; *P* = 0.030) but also the validation cohort (HR =4.075; 95% CI = 1.280–12.971; *P* = 0.017). Meanwhile, a higher UISS score also predicted poor OS of ccRCC patients in both two cohort groups (HR = 2.766; 95% CI = 1.139–6.713; *P* = 0.025 and HR =2.781; 95% CI = 1.059–7.300; *P* = 0.038, respectively) (Table 2). Taken together, these data indicate that FBXO11 upregulation was associated with poor ccRCC patient prognosis, and predicted worse OS in ccRCC patients after radical nephrectomy.

**Table 2:** Univariate and multivariate analyses for overall survival (OS) including pre-operatively known parameters as well as FBXO11 in 222 RCC patients treated with surgery

Overall Survival	Training cohort (150 patients)				Validation cohort (77 patients)			
	Univariate analysis		Multivariate analysis		Univariate analysis		Multivariate analysis	
	HR	*p* Value	HR	*p* Value	HR	*p* Value	HR	*p* Value
Gender	0.500 (0.219–1.142)	0.100	–	–	0.956 (0.428–2.135)	0.913	–	–
Age	1.664 (0.851–3.253)	0.136	–	–	1.554 (0.680–3.554)	0.296	–	–
Tumor size	1.606 (1.037–2.489)	**0.034**	1.276 (0.771–2.113)	0.343	1.461 (0.811–2.635)	**0.207**	1.267 (0.665–2.414)	0.471
FBXO11	2.817 (1.323–5.997)	**0.007**	2.381 (1.089–5.205)	**0.030**	5.740 (1.951–16.891)	**0.002**	4.075 (1.280–12.971)	**0.017**
Fuhrman grade	1.639 (1.045–2.568)	**0.031**	0.913 (0.533–1.563)	0.740	1.964 (1.206–3.198)	**0.007**	1.178 (0.708–1.960)	0.529
Pathologic stage	1.730 (1.089–2.747)	**0.020**	0.570 (0.243–1.338)	0.197	1.470 (0.971–2.226)	0.069		
Lymph node status	3.013 (0.723–12.557)	0.130			2.571 (0.873–7.577)	0.087		
Distant metastasis	1.732 (0.237–12.644)	0.588	–	–	3.246 (0.961–10.961)	0.058	–	–
Sarcomatoid	1.776 (0.628–5.028)	0.279	–	–	1.292 (0.303–5.499)	0.729	–	–
Type of surgery	1.366 (0.692–2.696)	0.369			1.351 (0.600–3.042)	0.468		
UISS score system	2.316 (1.489–3.601)	**0.001**	2.766 (1.139–6.713)	**0.025**	5.821 (2.604–13.015)	**0.001**	2.781 (1.059–7.300)	**0.038**
SSIGN score system	2.174 (1.240–3.812)	**0.007**	1.240 (0.503–3.061)	0.640	2.318 (1.404–3.826)	**0.001**	1.565 (0.774–3.163)	0.212

Bold values are considered statistically significant (*p* < 0.05)

### Nomogram of prognostic prediction based on FBXO11 expression in primary lesions

**Fig. 5:**
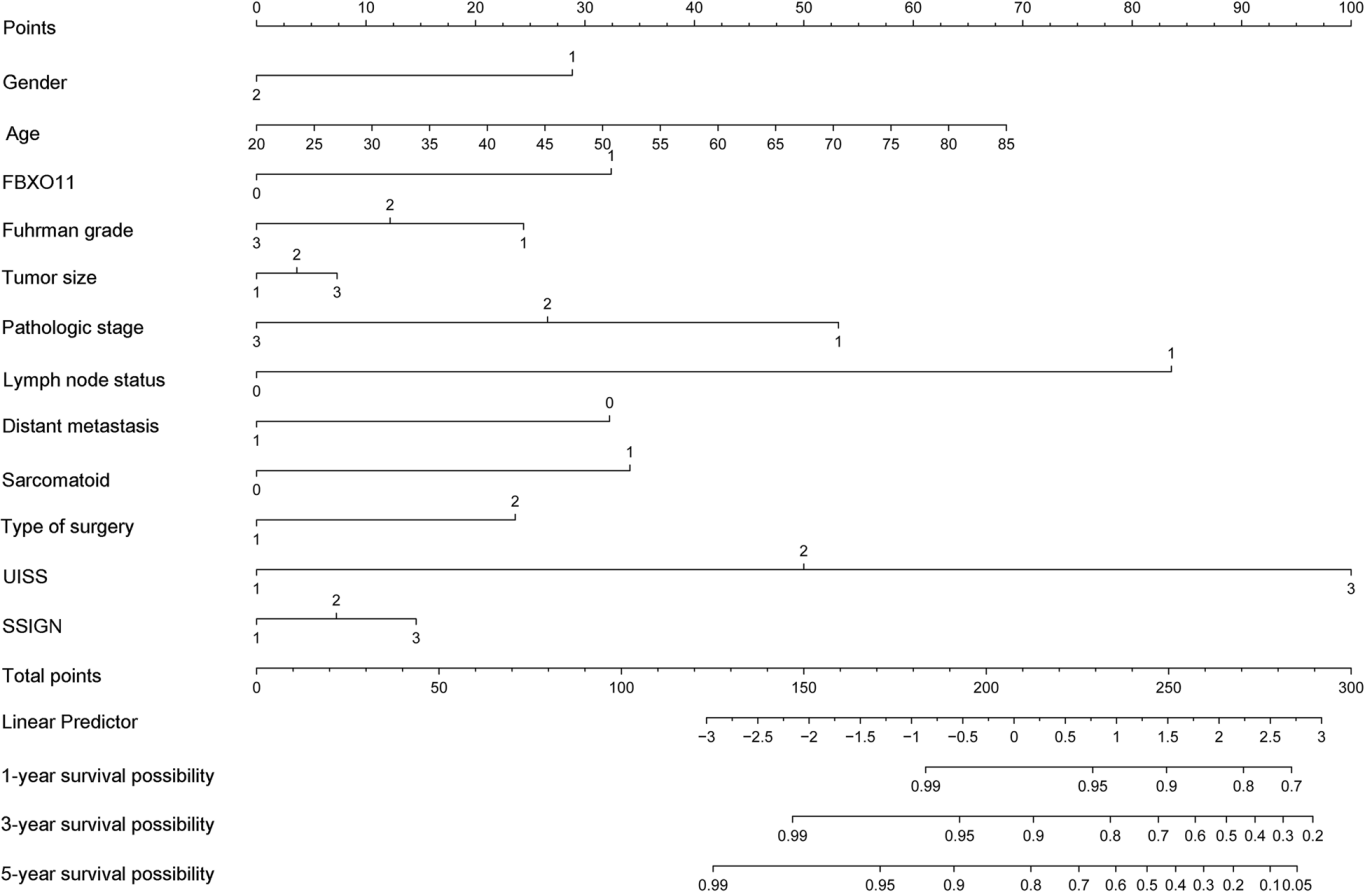
Nomogram model for the probability of 1-, 3- and 5- years overall survival (OS) predictions. The nomogram was used by summing the points based on the point designations corresponding to related factors including tumour-specific factors (size, SSIGN score, UISS score, differentiation, sarcomatoid, T stage), patient-specific factors (age, gender, type of surgery, lymph node status and distant metastasis) and FBXO11 expression

**Fig. 6:**
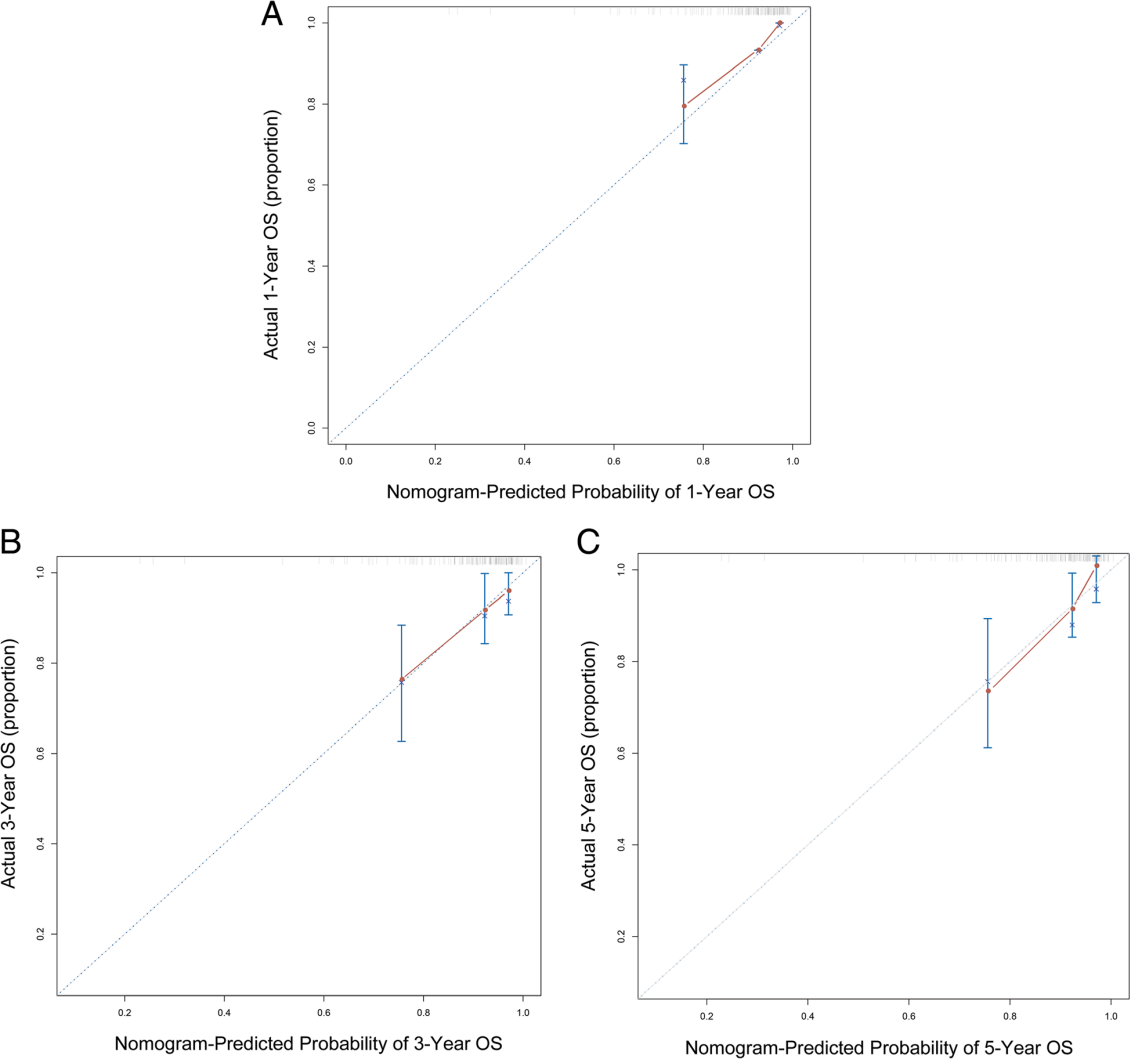
Calibration plots for predicting OS after nephrectomy . **a** Calibration plots for predicting OS at 1 year. **b** Calibration plots for predicting OS at 3 years. **c** Calibration plots for predicting OS at 5 years. The blue dotted line indicates the ideal nomogram; circles indicate the apparent predictive accuracy; blue X indicates the bootstrap-corrected estimates; vertical bars indicates the 95% CIs

**Fig. 7:**
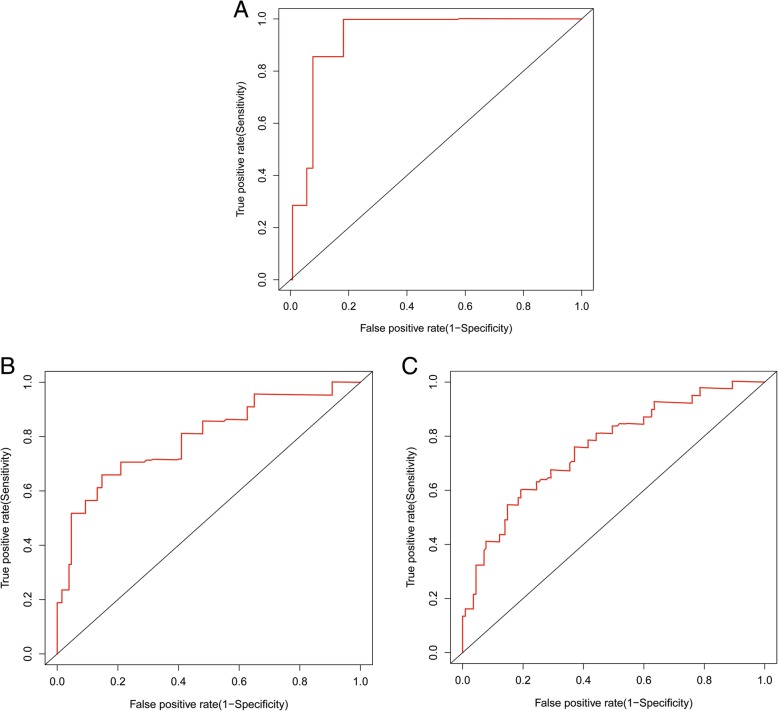
ROC curves of the 1-, 3- and 5-year nomograms of the training cohort after nephrectomy. **a** ROC curves of 1-year nomograms. **b** ROC curves of 3-year nomograms. **c** ROC curves of 5-year nomograms. The red lines represent nomogram-predicted overall survival rates, whereas the black lines represent AJCC TNM stage-predicted overall survival rates

To predict the 1-, 3- and 5-year OS rates of individual ccRCC patients, a novel nomogram model was established using the four significant prognostic factors in conjunction with age, gender, Fuhrman grade, tumour size, type of surgery, sarcomatoid, lymph node status and distant metastasis (Fig. 5). The calibration plots of the nomogram are shown for 1-, 3- and 5-year OS prediction (Fig. 6), of which the predictive probability of 3-year OS was very close to the actual 3-year OS. ROC curves of the 1-, 3- and 5-year nomograms are shown in Fig. 7a-c, with respective AUC values of 0.930, 0.792 and 0.757(Fig. 7).

### Extension of postoperative prognostic systems with FBXO11 expression

To establish a more sensitive model for predicting outcomes of patients with ccRCC, we combined FBXO11 expression with the UISS or SSIGN score and assessed their accuracy of survival. Incorporation of FBXO11 increased the predictive value of these three models, namely, when assessing OS: 0.703 versus 0.674 for the UISS score cohort, and 0.676 versus 0.598 for the SSIGN score cohort (Table 3). The elevated tendency of the C-index and the decreased trend of AIC suggest a better predictive accuracy. These results indicate that a combination of FBXO11 and conventional prognostic models could generate better predictive systems for ccRCC patient outcomes.

**Table 3:** Comparison of the prognostic accuracies of models for overall survival

Models	Training cohort (150 patients)		Validation cohort (77 patients)	
	C-index	AIC	C-index	AIC
FBXO11	0.628	343.01	0.692	185.42
UISS	0.674	339.38	0.728	179.34
UISS + FBOX11	0.703	336.58	0.780	175.13
SSIGN	0.598	345.66	0.665	190.02
SSIGN + FBOX11	0.676	340.92	0.757	178.22

## Discussion

Metastatic RCC presents the dual challenges of high mortality rates and significant management costs, thus highlighting the need to identify novel prognostic markers or therapeutic targets. Using the microarray datasets in ONCOMINE, we found that FBXO11 expression was lower in normal kidney tissues than in both ccRCC and pRCC, tissues and in primary cancers than in metastatic cancers. In addition, positive in situ FBXO11 expression was observed in 57.3% of the RCC tissues as opposed to 17.5% of the normal tissues. Furthermore, multivariate analysis showed that higher FBXO11 levels and larger tumour size were independent prognostic indicators of worse OS in patients. Taken together, these data suggest that high FBXO11 expression is associated with the degree of malignancy and poor patient prognosis for ccRCC. Based on its established oncogenic role in various cancer models, FBXO11 likely contributes to ccRCC progression.

The FBXO11 gene was first described as the vitiligo gene (VIT1) located on chromosome 2p21 and downregulated in vitiligo, a skin disorder characterized by the loss of melanocytes [19]. FBXO11 was identified as the substrate binding receptor in the Cullin RING E3 ubiquitin ligase 1 complex (also known as the SCF complex or the CRL1FBXO11 ubiquitin ligase) [20], a master regulator of cell cycle progression and genome stability [21]. FBXO11 stabilizes the CRL4Cdt2 substrates p21 and Set8 ubiquitylation and degradation of via Cdt2 (Cdc10-dependent transcript 2, also known as DTL/RAMP), and upregulates Set8 to promote Pr-Set7/Set8-mediated cellular migration [22]. Set8 accumulation in TGFβ-treated cells showed a decrease in Smad2 phosphorylation and N-cadherin induction [22]. This indicated that FBXO11-mediated Cdt2 degradation restrains the cellular response to TGFβ. Rossi et al. hypothesized that FBXO11-dependent degradation of Cdt2 controls the timing of cell cycle exit and differentiation [23]. In addition, FBXO11 mediates the degradation of the EMT transcription factor Snail in breast cancer cells. FBXO11 can not only cause direct ubiquitination and degradation of their target proteins, but also indirectly inhibit their function. For example, it promotes Nedd8 conjugation to p53, which inhibits the latter’s function [24].

Correlation analysis showed that tumour differentiation status was positively correlated with FBXO11 expression, indicating a higher probability of FBXO11 overexpression in poorly or moderately differentiated tumours than in highly differentiated tumours [25]. FBXO11 can regulate tumour formation or progression via several processes, such as growth suppression, EMT and angiogenesis. FBXO11 gene silencing led to the development of diffuse large B-cell lymphoma (DLBCL) by targeting the degradation of Bcl-6 [26], while FBXO11 inactivation resulted in abnormal germinal-centre formation. This activity is related to FBXO11-mediated degradation of phosphorylated Bcl-6 during BCR engagement [27]. While FBXO11 promotes proliferation, migration and invasion of gastric cancer cells via PI3K/AKT pathway-mediated EMT [28], it acts as a tumour suppressor in lung and breast cancer by degrading SNAIL, via promotion of serine-11 phosphorylation by protein kinase D1 (PKD1), and inhibiting SNAIL-induced EMT [15]. Finally, the ubiquitin ligase activity of FBXO11 destabilizes and inhibits de novo synthesis of HIF-1a, thereby promoting the glioblastoma cell response to hypoxia and inhibiting angiogenesis [29, 30].

Mutations or deletions in FBXO11 have been observed in various neoplastic and non-neoplastic conditions. For example, the Jeff mouse model of chronic otitis media harbours a FBXO11 mutation [31], which interferes with TGF-βsignalling [32, 33]. FBXO11 is also a target of miR-21 [30] and miR-26a, and the latter inhibits hepatocellular carcinoma (HCC) cell proliferation and metastasis by suppressing FBXO11 [34]. It is a direct functional target of miR-621 in breast cancer, and high miR-621 levels enhance the sensitivity of breast cancer cells to paclitaxel and carboplatin by suppressing FBXO11 and enhancing p53 activity [14]. Taken together, these findings support the notion that FBXO11 has a strong oncogenic role in HCC and breast cancer.

We established a prognostic nomogram model to predict the 1-, 3- and 5-year OS rates of individual ccRCC patients on the basis of FBXO11 expression, T stage, UISS score and SSIGN score combined with age, gender, Fuhrman grade, tumour size, type of surgery, sarcomatoid status, lymph node status and distant metastasis. Calibration plots of the nomograms indicated that the predicted 3-year OS closely corresponded to the actual 3-year OS, indicating high accuracy, specificity and simplicity of the model, which makes it feasible for regular clinical use [35–38].

There are several limitations of this study. First, the Kaplan–Meier and Cox regression analyses were conducted on a limited cohort size [39]. Second, we did not include all clinico-pathological parameters, such as postoperative adjuvant therapy, because they were unavailable. All of the limitations could have introduced potential selection bias.

## Conclusion

FBXO11 expression is an independent predictor of poor OS in ccRCC patients, indicating its potential as a prognostic factor and therapeutic target. More large-scale multicentre studies on ccRCC patients with long-term follow-up are needed to validate our findings. In addition, the exact mechanistic basis and signalling pathways underlying FBXO11 activity in urinary cancer have to be elucidated.

## Data Availability

The datasets used and/or analysed during the current study are available from the corresponding author upon reasonable request.

## Abbreviations

AIC: Akaike information criterion
AUC: Area under the curve
ccRCC: Clear cell renal cell carcinoma
C-index: Concordance index
EMT: Epithelial-mesenchymal transition
HR: Hazard ratio
IHC: Immunohistochemistry
OS: Overall survival
pRCC: Papillary renal cell carcinoma
RCC: Renal cell carcinoma
SSIGN: Stage, Size, Grade and Necrosis
UISS: UCLA Integrated Staging System

## References

[1] Bhatt JR, Finelli A (2014). Landmarks in the diagnosis and treatment of renal cell carcinoma. Nat Rev Urol.

[2] CJD W, Klaassen Z, Bhindi B, Ye XY, Chandrasekar T, Farrell AM, Goldberg H, Boorjian SA, Leibovich B, Kulkarni GS, Shah PS, Bjarnason GA, DYC H, Satkunasivam R, Finelli A (2018). First-line systemic therapy for metastatic renal cell carcinoma: a systematic review and network meta-analysis. Eur Urol.

[3] Siegel RL, Miller KD, Jemal A (2018). Cancer statistics, 2018. CA Cancer J Clin.

[4] Huang Q, Sun Y, Ma X, Gao Y, Li X, Niu Y, Zhang X, Chang C. Androgen receptor increases hematogenous metastasis yet decreases lymphatic metastasis of renal cell carcinoma. Nat Commun. 2017;8(918). 10.1038/s41467-017-00701-6. 10.1038/s41467-017-00701-6 PMC5640635 29030639

[5] Grünwald V, Lin X, Kalanovic D, Simantov R (2016). Early tumour shrinkage: a tool for the detection of early clinical activity in metastatic renal cell carcinoma. Eur Urol.

[6] Bhindi Bimal, Thompson R. Houston, Lohse Christine M., Mason Ross J., Frank Igor, Costello Brian A., Potretzke Aaron M., Hartman Robert P., Potretzke Theodora A., Boorjian Stephen A., Cheville John C., Leibovich Bradley C. (2018). The Probability of Aggressive Versus Indolent Histology Based on Renal Tumor Size: Implications for Surveillance and Treatment. European Urology.

[7] Gu L, Li H, Wang Z, Wang B, Huang Q, Lyu X, Shen D, Gao Y, Fan Y, Li X, Xie Y, Du S, Liu K, Tang L, Peng C, Ma X, Zhang X (2018). A systematic review and meta-analysis of clinicopathologic factors linked to oncologic outcomes for renal cell carcinoma with tumor thrombus treated by radical nephrectomy with thrombectomy. Cancer Treat Rev.

[8] Zhang L, Wu B, Zha Z, Zhao H, Feng Y (2018). The prognostic value and clinicopathological features of sarcomatoid differentiation in patients with renal cell carcinoma: a systematic review and meta-analysis. Cancer Manag Res.

[9] Sejima T, Iwamoto H, Morizane S, Hinata N, Yao A, Isoyama T, Saito M, Takenaka A (2013). The significant immunological characteristics of peripheral blood neutrophil-to-lymphocyte ratio and Fas ligand expression incidence in nephrectomized tumor in late recurrence from renal cell carcinoma. Urol Oncol.

[10] Kroeger N, Zimmermann U, Burchardt M, Pantuck AJ (2015). Prognostication in localised renal cell carcinoma. Lancet Oncol.

[11] Díaz VM, de Herreros AG (2016). F-box proteins: keeping the epithelial-to-mesenchymal transition (EMT) in check. Semin Cancer Biol.

[12] Jin Y, Shenoy AK, Doernberg S, Chen H, Luo H, Shen H, Lin T, Tarrash M, Cai Q, Hu X, Fiske R, Chen T, Wu L, Mohammed KA, Rottiers V, Lee SS, Lu J (2015). FBXO11 promotes ubiquitination of the Snail family of transcription factors in cancer progression and epidermal development. Cancer Lett.

[13] Guan C, Lin F, Zhou M, Hong W, Fu L, Xu W, Liu D, Wan Y, Xu A (2010). The role of VIT1/FBXO11 in the regulation of apoptosis and tyrosinase export from endoplasmic reticulum in cultured melanocytes. Int J Mol Med.

[14] Xue J, Chi Y, Chen Y, Huang S, Ye X, Niu J, Wang W, Pfeffer LM, Shao ZM, Wu ZH, Wu J (2016). MiRNA-621 sensitizes breast cancer to chemotherapy by suppressing FBXO11 and enhancing p53 activity. Oncogene.

[15] Zheng H, Shen M, Zha YL, Li W, Wei Y, Blanco MA, Ren G, Zhou T, Storz P, Wang HY, Kang Y (2014). PKD1 phosphorylation-dependent degradation of SNAIL by SCF-FBXO11 regulates epithelial-mesenchymal transition and metastasis. Cancer Cell.

[16] Fan B, Zhang H, Jin H, Gai Y, Wang H, Zong H, Jin M, Yang H, Wan S, Zhu J, Xu S, Wang J, Yang D, Song X (2016). Is overexpression of Ki-67 a prognostic biomarker of upper tract urinary carcinoma? A retrospective cohort study and meta-analysis. Cell Physiol Biochem.

[17] Jones J, Otu H, Spentzos D, Kolia S, Inan M, Beecken WD, Fellbaum C, Gu X, Joseph M, Pantuck AJ, Jonas D, Libermann TA (2005). Gene signatures of progression and metastasis in renal cell cancer. Clin Cancer Res.

[18] Beroukhim R, Brunet JP, Di NA, Mertz KD, Seeley A, Pires MM, Linhart D, Worrell RA, Moch H, Rubin MA, Sellers WR, Meyerson M, Linehan WM, Kaelin WG, Signoretti S (2009). Patterns of gene expression and copy-number alterations in von-hippel Lindau disease-associated and sporadic clear cell carcinoma of the kidney. Cancer Res.

[19] Le PIC, Sarangarajan R, Zhao Y, Stennett LS, Brown TL, Sheth P, Miki T, Boissy RE (2001). ‘VIT1’, a novel gene associated with vitiligo. Pigment Cell Res.

[20] Willems AR, Schwab M, Tyers M (2004). A hitchhiker’s guide to the cullin ubiquitin ligases: SCF and its kin. Biochim Biophys Acta.

[21] Jackson S, Xiong Y (2009). CRL4s: the CUL4-RING E3 ubiquitin ligases. Trends Biochem Sci.

[22] Abbas T, Mueller AC, Shibata E, Keaton M, Rossi M, Dutta A (2013). CRL1-FBXO11 promotes Cdt2 ubiquitylation and degradation and regulates Pr-Set7/Set8-mediated cellular migration. Mol Cell.

[23] Rossi M, Duan S, Jeong YT, Horn M, Saraf A, Florens L, Washburn MP, Antebi A, Pagano M (2013). Regulation of the CRL4(Cdt2) ubiquitin ligase and cell-cycle exit by the SCF (Fbxo11) ubiquitin ligase. Mol Cell.

[24] Abida WM, Nikolaev A, Zhao W, Zhang W, Gu W (2007). FBXO11 promotes the Neddylation of p53 and inhibits its transcriptional activity. J Biol Chem.

[25] Frescas D, Pagano M (2008). Deregulated proteolysis by the F-box proteins SKP2 and beta-TrCP: tipping the scales of cancer. Nat Rev Cancer.

[26] Duan S, Cermak L, Pagan JK, Rossi M, Martinengo C, di CPF, Chapuy B, Shipp M, Chiarle R, Pagano M (2012). FBXO11 targets BCL6 for degradation and is inactivated in diffuse large B-cell lymphomas. Nature.

[27] Schneider C, Kon N, Amadori L, Shen Q, Schwartz FH, Tischler B, Bossennec M, Dominguez-Sola D, Bhagat G, Gu W, Basso K, Dalla-Favera R (2016). FBXO11 inactivation leads to abnormal germinal-center formation and lymphoproliferative disease. Blood.

[28] Sun C, Tao Y, Gao Y, Xia Y, Liu Y, Wang G, Gu Y (2018). F-box protein 11 promotes the growth and metastasis of gastric cancer via PI3K/AKT pathway-mediated EMT. Biomed Pharmacother.

[29] Ju UI, Park JW, Park HS, Kim SJ, Chun YS (2015). FBXO11 represses cellular response to hypoxia by destabilizing hypoxia-inducible factor-1α mRNA. Biochem Biophys Res Commun.

[30] Yang CH, Pfeffer SR, Sims M, Yue J, Wang Y, Linga VG, Paulus E, Davidoff AM, Pfeffer LM (2015). The oncogenic microRNA-21 inhibits the tumor suppressive activity of FBXO11 to promote tumorigenesis. J Biol Chem.

[31] Hardisty-Hughes RE, Tateossian H, Morse SA, Romero MR, Middleton A, Tymowska-Lalanne Z, Hunter AJ, Cheeseman M, Brown SD (2006). A mutation in the F-box gene, Fbxo11, causes otitis media in the Jeff mouse. Hum Mol Genet.

[32] Tateossian H, Hardisty-Hughes RE, Morse S, Romero MR, Hilton H, Dean C, Brown SD (2009). Regulation of TGF-beta signalling by Fbxo11, the gene mutated in the Jeff otitis media mouse mutant. Pathogenetics.

[33] Tateossian H, Morse S, Simon MM, Dean CH, Brown SD (2015). Interactions between the otitis media gene, Fbxo11, and p53 in the mouse embryonic lung. Dis Model Mech.

[34] Ma Y, Deng F, Li P, Chen G, Tao Y, Wang H (2018). The tumor suppressive miR-26a regulation of FBXO11 inhibits proliferation, migration and invasion of hepatocellular carcinoma cells. Biomed Pharmacother.

[35] Balachandran VP, Gonen M, Smith JJ, DeMatteo RP (2015). Nomograms in oncology: more than meets the eye. Lancet Oncol.

[36] Shariat SF, Karakiewicz PI, Suardi N, Kattan MW (2008). Comparison of nomograms with other methods for predicting outcomes in prostate cancer: a critical analysis of the literature. Clin Cancer Res.

[37] Iasonos A, Schrag D, Raj GV, Panageas KS (2008). How to build and interpret a nomogram for cancer prognosis. J Clin Oncol.

[38] Huang YQ, Liang CH, He L, Tian J, Liang CS, Chen X, Ma ZL, Liu ZY (2016). Development and validation of a Radiomics nomogram for preoperative prediction of lymph node metastasis in colorectal Cancer. J Clin Oncol.

[39] Parker AS, Eckel-Passow JE, Serie D, Hilton T, Parasramka M, Joseph RW, Wu KJ, Cheville JC, Leibovich BC (2014). Higher expression of topoisomerase II alpha is an independent marker of increased risk of cancer-specific death in patients with clear cell renal cell carcinoma. Eur Urol.

